# Challenges in Managing Undiagnosed Prenatal Sacrococcygeal Teratoma—Case Report and Literature Review

**DOI:** 10.3390/jcm15114131

**Published:** 2026-05-27

**Authors:** Jagoda Langiewicz, Olga Wiśniewska, Jakub Rzepka, Michał Michalczyk, Marzena Michalak-Kloc, Marcin Polok, Rafał Rzepka

**Affiliations:** 1 Department of Gynecology and Obstetrics, University of Zielona Gora, Zyty 26, 65046 Zielona Gora, Poland; 2 Poznan University of Medical Sciences, 61701 Poznań, Poland; 3 Department of Pediatrics, University of Zielona Gora, Zyty 26, 65046 Zielona Gora, Poland; 4 Department of Pediatric Surgery and Urology, University of Zielona Gora, Zyty 26, 65046 Zielona Gora, Poland

**Keywords:** sacrococcygeal teratoma, fetal tumor, germ cell tumor, obstetric management

## Abstract

**Background/Objectives**: Teratomas of the sacrococcygeal region are rare, but the most common tumors found in fetuses. They develop from the three germ layers—mesoderm, ectoderm, and endoderm—and occur at a rate of 1 in 27,000 to 1 in 40,000, with a fourfold higher incidence in female fetuses. 63.9–74% of sacrococcygeal teratomas are detected prenatally, most often in the second trimester. **Methods**: This study reports the case of a woman in her second pregnancy at 29 weeks and 2 days gestation who was incidentally diagnosed with tumor-like lesion in the sacrococcygeal region of fetus. The clinical situation required the pregnancy to be delivered by emergency cesarean section, and the tumor was surgically removed within the first few days of life. The lesion was finally diagnosed as an immature teratoma, and appropriate management was initiated, resulting in stabilization of the child’s general condition and proper development. **Results:** Detailed imaging and characterization of the lesion are essential for determining the appropriate management and minimizing foreseeable obstetric and neonatal complications. Fetal echocardiography in cases of suspected teratoma in the sacrococcygeal region is essential for identifying life-threatening risk factors and influences the planning of further management. The choice of treatment depends on the clinical situation; among intrauterine interventions and pharmacological therapy, it has been demonstrated that surgical removal of the lesion within the first days of life reduces the risk of recurrence. **Conclusions**: In any case where lesion such as tumor of the sacrococcygeal region of fetus is suspected, the pregnant woman should be managed at a tertiary care center to ensure multidisciplinary care involving obstetricians, neonatologists, pediatric surgeons, and oncologists. This study provides a review of the literature on methods of diagnosis and treatment of sacrococcygeal teratomas in fetuses. It emphasizes the importance of accurate diagnosis and prenatal care in such cases and their impact on further management.

## 1. Introduction

Sacrococcygeal teratoma (SCT), although rare, is the most common germ cell tumor occurring in fetuses [1,2]. It is characterized by the presence of tissue components derived from multiple germ layers [3].

Teratomas in neonates most commonly occur in the sacrococcygeal region, with less frequent involvement of the mid-pharyngeal region, brain, mediastinum, abdominal cavity, gonads, retroperitoneal space, and pericardium [4].

The incidence of sacrococcygeal teratoma varies. The reported prevalence of sacrococcygeal teratoma ranges from approximately 1 in 27,000 in Denmark to as high as 1 in 40,000 in other studies [5,6]. Sacrococcygeal teratoma occurs more frequently in females, with a female-to-male ratio of 4.6:1. With routine prenatal ultrasound, 44–74% of cases can be detected before birth [6,7]. Ultrasound is the primary prenatal diagnostic method for SCT and should assess tumor size and growth rate, ratio of solid-to-cystic components, vascularization, and the presence of polyhydramnios, fetal hydrops, and cardiac abnormalities [8,9,10].

A 26-year retrospective cohort study from Denmark reported that the majority of tumors were composed of mature tissue (48%), while 34% contained immature elements and 18% were malignant [6].

Most cases occur sporadically, although 16% are associated with congenital anomalies, including Currarino syndrome [6,11,12].

Perinatal management of SCT should be planned at a tertiary referral center with a multidisciplinary team [2,13,14]. Although most fetuses diagnosed with SCT are delivered by cesarean section, vaginal delivery may be considered in selected cases [10,15,16].

Perinatal management of SCT is risk-adapted and depends on gestational age and fetal condition, ranging from intrauterine interventions before 27 weeks through ex utero intrapartum treatment (EXIT procedure) to early postnatal radical resection with coccygectomy when the EXIT procedure is not pursued [5,17,18,19,20,21,22,23,24].

The reported recurrence rate of SCT is 10–16%, with most recurrences occurring within the first 3 years after surgery; however, late recurrences have been documented up to 20 years after primary resection [6,25,26,27].

Although long-term follow-up is recommended after SCT resection, there is currently no standardized surveillance protocol, which may delay the detection of recurrence and late complications [25]. Follow-up strategies also differ between mature and immature teratomas, yet both include AFP monitoring and regular imaging [26,28].

This study presents an unusual case of an undiagnosed prenatal sacrococcygeal teratoma that led to preterm birth, in which a rare variant of the lesion was detected, followed by a review of the literature on the diagnosis and treatment of such lesions.

## 2. Case Report

A 28-year-old pregnant woman (G2 P2, 29 + 2 weeks of gestation) was admitted to the Obstetrics and Gynecology Department, transferred from a lower-level hospital with suspected myelomeningocele due to a mass in the sacral region visualized on ultrasound and polyhydramnios. The patient presented with symptoms of vaginal spotting and lower abdominal pain, which subsided. The course of the pregnancy to date had been uncomplicated, and the pregnancy had been under regular outpatient care. Prenatal tests were not performed due to the patient’s lack of consent. The patient’s medical and family history was unremarkable for chronic, neoplastic, or genetic diseases, except for nicotine use. The first pregnancy was uncomplicated and resulted in a spontaneous vaginal delivery of a healthy neonate at term.

**Figure 1:**
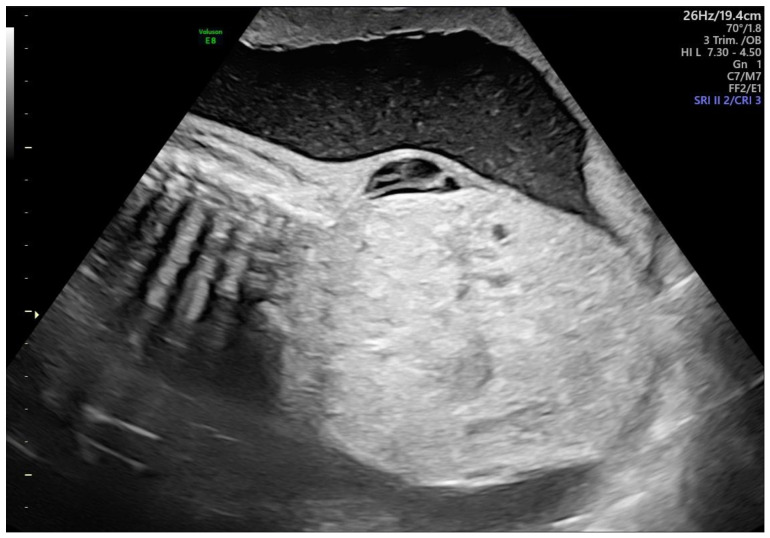
Prenatal ultrasound demonstrating a massive sacrococcygeal teratoma.

The patient was admitted to the High-Risk Pregnancy Unit for further diagnostic work-up. Ultrasonography revealed a single fetus in cephalic presentation with a sacrococcygeal mass measuring approximately 11 × 11 cm (Figure 1). The estimated tumor volume was 657.3 mL. Polyhydramnios was absent, with a maximum vertical pocket (MVP) of 6.9 cm and an estimated fetal weight (EFW) of 1366 g. Tumor to fetal ratio was 0.48. No signs of hydrops, including ascites, pleural effusion, pericardial effusion, or generalized skin edema, were observed. There was also no evidence of cardiomegaly or fetal cardiac failure. Placental thickness was 31 mm. Preterm premature rupture of membranes (PPROM) was diagnosed during the gynecological examination, which also revealed a shortened cervix with 2 cm dilation and an engaged fetal head; no vaginal bleeding was noted. Given the presence of meconium-stained amniotic fluid and active uterine contractions, antenatal corticosteroids were administered. Due to the urgency of the case after the transfer given the presence of meconium-stained amniotic fluid and active uterine contractions and the lack of earlier diagnosis, some measurements recommended in fetal sacrococcygeal mass management could not be obtained. 

**Figure 2:**
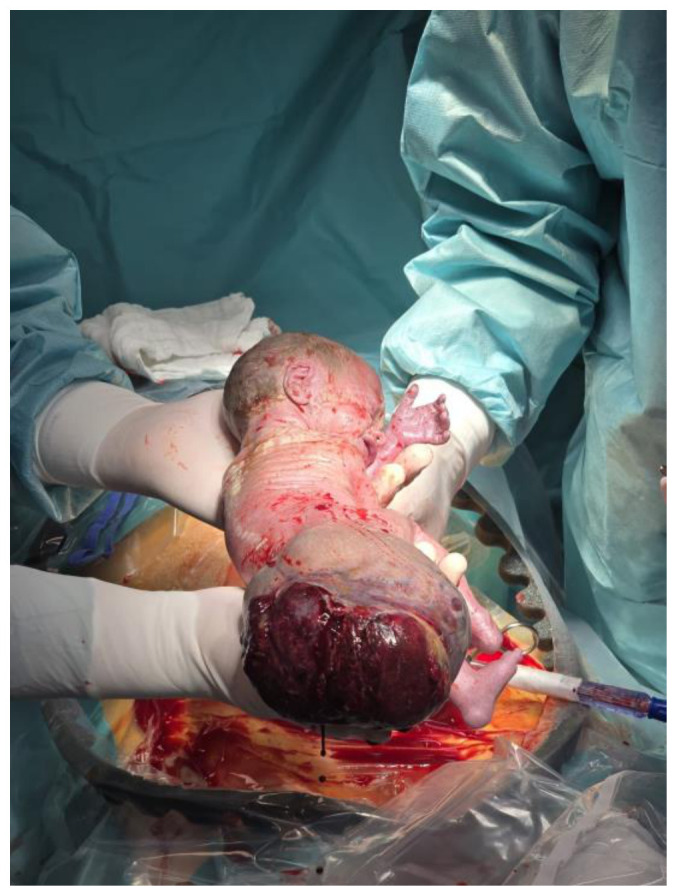
A newborn with a tumor in the sacrococcygeal region directly after delivery.

On the day of admission, an emergency cesarean section was performed due to suspected chorioamnionitis, the presence of a fetal sacrococcygeal mass, and active labor. The neonate’s clinical condition at birth was critical, with Apgar scores of 6, 6, 4, and 4 at 1, 3, 5, and 10 min, respectively. Initial laboratory findings were as follows: arterial pH 7.39, base excess (BE) −1.4, hematocrit 30.4%, and hemoglobin 9.9 g/dL. The presence of meconium-stained amniotic fluid was confirmed. Physical examination revealed a sacral mass with skin denudation over one-third of its surface and active external hemorrhage, which was managed with a sterile dressing. The birth weight, excluding the tumor mass, was approximately 1100 g. At 4 min of life, the neonate was intubated due to marked pallor and transferred to the Neonatal Intensive Care Unit (NICU). The infant’s hemodynamic instability necessitated urgent red blood cell transfusion, endotracheal surfactant administration, and broad-spectrum antibiotic therapy. During the NICU stay, the patient developed progressive edema and oliguria. Laboratory abnormalities, including thrombocytopenia, anemia, and coagulopathy, required multiple transfusions of platelets, fresh frozen plasma (FFP), and packed red blood cells. Following a pediatric surgical consultation, the tumor was secured with hemostatic dressings. Due to the patient’s critical condition, surgical intervention was deferred until clinical stabilization was achieved (Figure 2).

**Figure 3:**
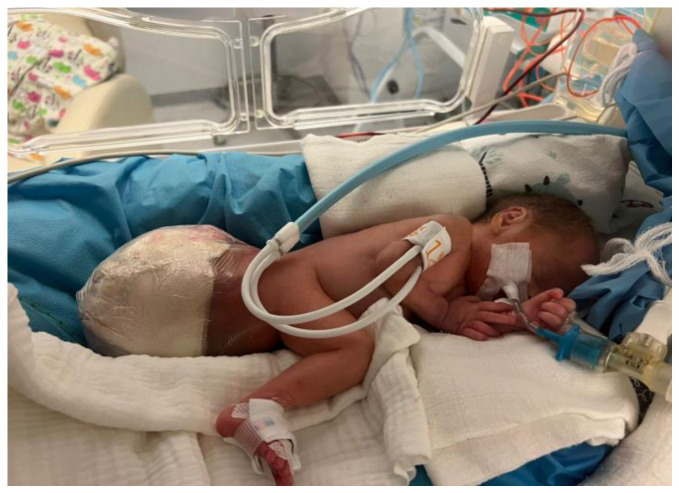
Neonatal Intensive Care Unit stay.

Echocardiography performed on day 1 of life revealed a patent foramen ovale (PFO) and ductus arteriosus (PDA), which are considered physiological findings in the early postnatal period. Notably, the neonate exhibited no clinical signs of heart failure or circulatory insufficiency. Cranial ultrasonography demonstrated high-resistance flow within the middle cerebral artery(Figure 3).

On the same day, an abdominal and pelvic computed tomography (CT) scan (including non-contrast and venous phases) identified a voluminous, heterogeneous mixed solid-cystic mass measuring approximately 120 × 85 × 120 mm (AP × TR × CC). The lesion, which originated within the pelvis and extended inferiorly beyond the body contours, was characterized by an extensive network of pathological neovascularization. Due to its significant dimensions, the precise anatomical origin of the tumor could not be definitively established. In addition to the intrinsic intratumoral vessels, there was a strong suspicion of accessory vascular supply arising from the left common and internal iliac arteries. Furthermore, a heterogeneous hepatic parenchymal pattern was noted, suggesting the possibility of secondary metastatic lesions.

**Figure 4:**
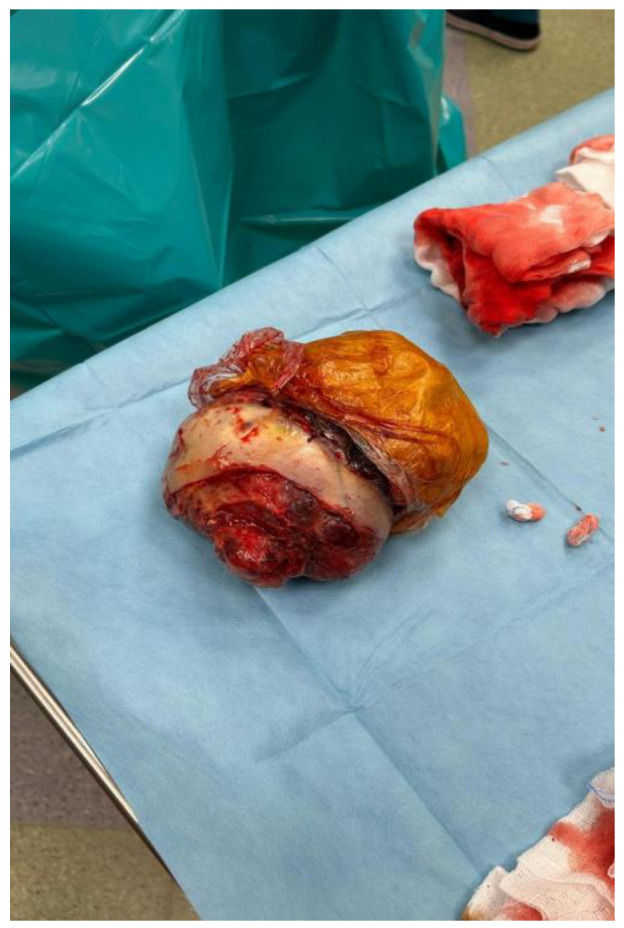
A tumor in the sacrococcygeal region excised during surgery.

Following clinical stabilization, surgical resection was performed on day 3 of life under general endotracheal anesthesia in the prone position. Intraoperatively, the tumor was progressively dissected from the overlying skin and gluteal musculature. Following the insertion of an 18 Fr Foley catheter into the rectum to facilitate identification, the mass was meticulously separated from the rectal wall, to which it was firmly adherent over a significant segment. Total excision of the 570 g tumor along with the coccyx (en bloc resection) was achieved, and the specimen was submitted for histopathological evaluation (Figure 4). Subsequent reconstruction of the gluteal muscles and the anorectal region was performed, including the excision of redundant skin and subcutaneous tissue. The procedure required intraoperative red blood cell transfusion. Postoperatively, the neonate was maintained under sedation until day 7, followed by extubation and transition to non-invasive positive pressure ventilation (NIPPV). Antibiotic therapy was continued through day 9.

On the third postoperative day, a follow-up abdominal and pelvic CT (pre- and post-contrast) revealed an enlarged left inguinal lymph node with increased enhancement, for which ultrasound re-evaluation was recommended. No further lesions suggestive of distant metastases were identified. Given the immediate postoperative period, the surgical radicality could not be definitively assessed. Magnetic resonance imaging (MRI) performed on postoperative day 10 demonstrated expected postoperative changes alongside an irregular, predominantly cystic lesion measuring 10 × 6 × 7 mm (TR × AP × CC) in the presacral space. This lesion exhibited peripheral enhancement, more pronounced on the right side, and was interpreted as consistent with residual tumor. On day 13 of life, a surgical follow-up confirmed primary intention healing of the postoperative wound. Enteral feeding was well tolerated, and bowel function remained normal. Histopathological examination revealed a tumor measuring 13 × 10 × 6.5 cm, partially covered by a 25 × 4.5 cm skin flap. Surgical margins were inked for orientation. Gross examination on section showed a cream-colored mass with cystic components, areas of hemorrhage, and necrosis. The microscopic appearance was consistent with an immature teratoma. Given that the surgical margin coincided with the tumor capsule, the oncological radicality remained uncertain, necessitating a second-opinion pathological review. On day 34 of life, the final diagnosis of grade 3 immature teratoma was confirmed. Following oncological consultation, a surveillance protocol was initiated, including serial monitoring of tumor markers (AFP, β-hCG, LDH) and periodic ultrasonography. The clinical data were reviewed by the national pediatric oncology coordinator. In light of the steadily declining AFP levels and the absence of metastatic spread to the liver, lungs, or abdominal cavity, systemic pharmacological therapy was not indicated at that time. During the stay in the Neonatal Department, feeding with mother’s milk was gradually introduced from day 21 of life until full feeds were achieved. From day 49 of life, the infant was breathing spontaneously with passive oxygen therapy. Routine ultrasound diagnostics and ophthalmologic consultations were performed. Tumor markers showed a downward trend, the postoperative wound healed properly, and appropriate weight gain was observed. The child was discharged on day 81 of life in good general condition, weighing 2475 g, with recommendations for monitoring AFP, β-hCG, LDH levels and ultrasound every 2 weeks, and MRI of the pelvis, abdomen, and spinal canal monthly under the care of the Department of Pediatrics with Hematology and Oncology Unit. The first scheduled MRI was postponed due to an upper respiratory tract infection and anesthesiology disqualification.

At 3 months of age, follow-up CT of the chest, abdomen, and pelvis under general anesthesia (the MRI scan was not available on the scheduled date due to equipment maintenance) showed no detectable distant metastases. At the follow-up examination, the child was in good general condition, with normal urination and bowel movements and appropriate weight gain (+25 g/day). No abnormalities were found on physical examination. Laboratory tests showed a continued decrease in tumor markers: AFP initially >121,000.0 ng/mL, currently 232.0 ng/mL; β-hCG initially 0.54 mIU/mL, currently <0.20 mIU/mL; LDH increased from 270 U/L to 351 U/L. The child remains under ongoing specialist care (ophthalmologist, audiologist, neonatologist) with recommendations for continued monitoring of tumor markers and imaging diagnostics (Table 1).

## 3. Discussion

### 3.1. Classification

SCT is most commonly classified according to the Altman system, developed by the Surgical Section of the American Academy of Pediatrics:

Type I—a predominantly external tumor with a minimal presacral component;

Type II—an external tumor with a significant internal component extending into the pelvis;

Type III—a mainly internal tumor with a small external portion;

Type IV—an entirely presacral tumor without any external component [29].

Altman types III and IV are associated with a higher frequency of long-term complications, including anorectal dysfunction, urinary dysfunction, and lower limb motor dysfunction [28].

From a histological perspective, SCTs can be classified as mature (48%), immature (34%), or malignant (18%). In neonates, the vast majority of lesions are benign [6,8].

### 3.2. Prenatal Evaluation

Congenital SCTs are typically diagnosed prenatally in 63.9–74% of cases [7,30]. They are typically detected during second-trimester ultrasound, and less frequently in the third trimester [31]. However, there are case reports of SCT being detected as early as the first trimester [32]. The majority of SCTs identified prenatally are classified as Altman Type I or II [33].

Ultrasound is the primary diagnostic method in diagnosing SCT prenatally. It should be performed with particular focus on:

- Tumor size and growth rate;
- The ratio of solid to cystic components;
- Degree of vascularization;
- Presence of polyhydramnios;
- Signs of fetal hydrops;
- Fetal echocardiography [8,9,10].

Tumors with a predominantly solid component—being the least common, highly vascularized, and rapidly growing—are associated with a higher rate of perinatal complications [9,10].

A high degree of tumor vascularization and multiple arteriovenous shunts can lead to volume overload of the fetal circulatory system, resulting in tachycardia and cardiomegaly, which can progress to heart failure, hydrops fetalis, and even intrauterine fetal demise, highlighting the importance of performing fetal echocardiography [8,34].

The tumor-to-fetus volume ratio is another key prognostic indicator. A TFR exceeding 0.12 prior to 24 weeks of gestation is associated with an unfavorable prognosis [35].

Tumor growth rate assessed by ultrasound seems to be an important prognostic factor. Vinit et al. reported that a tumor increase exceeding 7 mm per week is associated with adverse perinatal outcomes [36].

Three-dimensional ultrasonography is particularly useful in the assessment of congenital SCTs, allowing for a more detailed evaluation of sacral bone and pelvic structure involvement [37]. Magnetic resonance imaging (MRI) may also prove useful in the evaluation of SCTs, as it enables detailed assessment of tumor infiltration into the spinal canal and aids in differentiating other pathologies in the sacrococcygeal region. This imaging modality is particularly important when SCT is detected during the second half of pregnancy or in patients with maternal obesity, where ultrasound visualization may be limited [38]. Li et al. reported a significantly higher sensitivity of MRI over ultrasonography, reaching up to 98%; however, larger studies on broader patient populations are needed. An additional advantage of MRI over ultrasound is that its accuracy is not affected by gestational age, amniotic fluid volume, or fetal position [39].

### 3.3. Perinatal Complications

Prenatal diagnosis is critically important, as it enables close monitoring of fetal heart failure and cardiomegaly, facilitates delivery planning at a tertiary care center, and ensures adequate preparation of the surgical team. Without prenatal recognition, the risk of severe neonatal hemodynamic instability and massive hemorrhage is noticeably elevated [2,13,31,40,41]. The most significant prenatal complications include polyhydramnios, preterm birth, hydrops fetalis, cardiomegaly, fetal heart failure, and intrauterine fetal demise [30,42]. Studies show that 81% of patients experience at least one obstetric complication, with 72.7% delivering before 37 weeks and 29.1% before 32 weeks of gestation [31,43].

The most significant predictors of poor prenatal outcome are hydrops fetalis (OR: 21.0), cardiomegaly (OR: 10.3), highly vascularized tumors, solid tumor morphology, and placentomegaly [9,44]. The overall prenatal and perinatal mortality, excluding pregnancy terminations, ranges from 11 to 13% [7,44].

### 3.4. Perinatal Management

Perinatal management of fetuses diagnosed with congenital sacrococcygeal teratoma (SCT) should be planned at a high-level referral center equipped with a multidisciplinary team including specialists in obstetrics, neonatology, and pediatric surgery [2,13,14].

A key aspect of delivery planning is the risk stratification of patients, as well as the assessment of tumor size, vascularization, growth rate, and overall fetal condition, as these factors can significantly influence the risk of perinatal complications [7,45,46]. Factors defining high-risk SCTs include high-output heart failure, hydrops fetalis, tumor hemorrhage, and a tumor-to-fetus ratio (TFR) > 0.12. Patients without these features should be classified as low-risk SCTs [5].

In patients with small tumors, management of low-risk SCTs typically involves planned cesarean section after 36 weeks of gestation [10]. However, expectant management with vaginal delivery may be considered for fetuses with small tumors and an otherwise uncomplicated pregnancy [15,16]. Polish guidelines recommend cesarean delivery when the tumor diameter exceeds 10 cm, although other sources suggest that an external tumor diameter of 5 cm is sufficient to indicate planned cesarean section. Large, exophytic tumors can mechanically obstruct vaginal delivery and increase the risk of dystocia [5,17,40].

In fetuses presenting with high-risk factors after 27 weeks of gestation, the preferred management is emergency cesarean section if maternal complications occur, or an EXIT procedure—which involves partial delivery of the fetus while maintaining placental support for immediate tumor resection—in the absence of maternal complications [5].

The advantages of the EXIT procedure over traditional surgery seem to include a reduced risk of fetal asphyxia caused by failed intubation, which is more likely due to the specific surgical positioning required during SCT resection. Additionally, placental blood flow can help compensate for fetal blood loss during tumor removal. However, due to the rarity and unpredictable nature of SCT, EXIT has only rarely been successfully applied [18]. The EXIT procedure is also associated with significantly greater maternal blood loss [19]. Therefore, conventional surgical resection should still be considered.

In fetuses presenting with high-risk factors before 27 weeks of gestation, the preferred management is intrauterine intervention aimed at intratumoral ablation using electrocautery, radiofrequency, or laser energy, as well as occlusion of tumor vessels via laser or sclerotherapy [5,17,20,21].

Attempts to improve fetal cardiac inotropy can be undertaken through pharmacological therapy with digoxin or by partial tumor resection (debulking). Amnioreduction or aspiration of tumor contents—particularly in predominantly cystic lesions—can be useful in cases of threatened preterm labor caused by polyhydramnios or excessive uterine distension due to the tumor [31].

It should be noted that data supporting specific management approaches are somewhat inconsistent across different publications. Therefore, the decision regarding the mode of delivery should be made on an individual basis, taking into account imaging findings and ongoing assessment of fetal well-being [22].

If the EXIT strategy is not pursued, surgical resection should be performed early, ideally within the first days of life, which helps to minimize the risk of tumor recurrence [23]. Tumor resection should be as radical as possible and must always include en bloc coccygectomy to minimize the risk of recurrence [24].

### 3.5. Prognosis and Mortality

Mortality associated with SCT is approximately 3% [25].

Factors associated with poor prognosis include:

- Hydrops fetalis;
- Large, rapidly growing solid tumors;
- Preterm birth;
- Immature or malignant histology;
- Incomplete tumor resection;
- Surgical complications [7,28,44,47,48].

Interestingly, mortality is higher in fetuses diagnosed prenatally compared to those diagnosed postnatally, likely because smaller tumors, which are often missed prenatally, tend to have a more favorable prognosis [9,34]. It is estimated that in cases of prenatal diagnosis, the mortality rate ranges from 30 to 50% [49].

The presence of a Yolk Sac Tumor (YST) component significantly worsens prognosis and increases the risk of malignant recurrence. In the study by Johnston et al., even microscopic foci of YST within mature teratomas raised the recurrence risk to 56%, compared to 4% in purely mature teratomas [50].

### 3.6. Long-Term Complications

The most common long-term complications following resection of congenital SCT are bladder and bowel dysfunction, affecting 20–36% of patients [28,51]. Anorectal dysfunction (constipation, soiling) occurs in 17–22% of children, urinary disorders (neurogenic bladder, difficulty with bladder emptying) in 7–24%, and lower limb motor impairments in 4–10% [6,28,52,53].

Risk factors for functional complications include preterm birth, large tumor size, Altman type III–IV (particularly type IV, with 60% experiencing urinary disorders), incomplete resection, surgical complications, and immature or malignant histology [54]. Endopelvic tumors causing prenatal urinary tract compression are associated with neurogenic bladder. These patients also more frequently experience bowel dysfunction [52].

Despite a substantial incidence of dysfunction, quality of life is reported as good or very good in the majority of patients (56% in the 4–7-year age group, 90% in 8–17 years, and 67% in adults) [53].

### 3.7. Recurrence and Follow-Up

Recurrence of SCT is observed in 10–16% of patients, typically within the first 3 years post-surgery, although late recurrences have been documented even 20 years after the primary resection [6,25,26,27]. Notably, 36–50% of SCT recurrences are malignant, with the yolk sac tumor component being the most frequent [26,27]. Mature teratomas can recur as malignant tumors—one-third of recurrences show progression toward immaturity or malignancy compared to the primary tumor [48].

Risk factors for SCT recurrence include incomplete resection (OR 6.54), malignant histology (OR 12.83) or immature histology (OR 5.74), Altman type II–III, tumor spillage during surgery, and surgical complications [27,48,55].

Recurrence-free survival is 93.7% at 1 year and 88.8% at both 3 and 5 years [25]. The timing of diagnosis, whether prenatal or postnatal, does not seem to significantly influence recurrence patterns [25].

### 3.8. Follow-Up Recommendations

Srivatsa et al. highlight the issue of a lack of standardized long-term follow-up, which may lead to delayed detection of recurrence and late complications, significantly affecting prognosis [25]. Gil et al. even point out variability in practice within a single center [56].

Currently, for both mature and immature tumors, a minimum of 3 years of close follow-up is generally recommended, including imaging studies, tumor markers such as alfa-fetoprotein (AFP), and clinical examination [25]. For tumors with high-risk features (immature, malignant, or containing microscopic YST foci), follow-up for 6–10 years or longer appears appropriate; however, there is currently insufficient evidence to establish a standardized surveillance protocol [26,28].

Malignant recurrences are primarily detected through rising AFP levels, while mature recurrences are identified mainly via MRI and ultrasound imaging [50,57]. The sensitivity of AFP for detecting malignant SCT recurrences is as high as 96% [58].

While MRI and ultrasonography are widely used for the initial evaluation of SCTs, there is limited evidence regarding the most effective imaging modality for detecting recurrence. Ultrasound is commonly employed due to its accessibility and the absence of sedation requirements, whereas MRI provides superior visualization of internal anatomy and surrounding structures [57].

## 4. Conclusions

This study highlights the importance of prenatal diagnosis and care when a tumor-like lesion is detected in the sacrococcygeal region of the fetus. First and foremost, thorough ultrasound diagnosis—as the most accessible and minimally invasive examination—not only allows for the detection of lesions or abnormalities but also enables the determination of further management and prognosis based on the analysis of specific factors. The diagnosis of teratoma in the sacrococcygeal region still remains a challenge for present-day medicine, requiring multidisciplinary care and access to appropriate diagnostic and monitoring tools. Although magnetic resonance imaging is the recommended method for diagnosis and continuing follow-up, access to this test is still limited.

In the presented case, the absence of a comprehensive diagnostic pathway resulted from the incidental detection of the lesion during the evaluation of acute maternal symptoms. Crucially, the lack of prenatal diagnosis precluded the implementation of intrauterine therapy, which directly contributed to the onset of preterm labor. Furthermore, the lack of early detection necessitated deferring surgical intervention until clinical stabilization was achieved. The clinical scenario required rapid, decisive action to balance maternal and fetal well-being against the risk of imminent complications. Despite multiple adverse prognostic factors—including prematurity, significant tumor volume (leading to subtotal resection), and the presence of a rare histological variant (grade 3 immature teratoma)—follow-up assessments have demonstrated no typical dysfunction of the excretory or urinary systems, with no current indication for adjuvant pharmacological therapy. Although the delayed excision may potentially increase the long-term risk of recurrence, current follow-up confirms age-appropriate development and a consistent downward trend in tumor markers. Nevertheless, continued multidisciplinary surveillance remains mandatory.

## Figures and Tables

**Table 1:** Timeline of diagnostic evaluation, surgical treatment, and follow-up.

Timeframe	Clinical Findings and Management Details
**29 + 2 Weeks Gestation**	**Prenatal Referral and Admission to High-Risk Pregnancy Unit** Referred to a tertiary center for suspected myelomeningocele or fetal sacrococcygeal mass. Complications noted: PPROM, shortened cervix with 2 cm dilation and an engaged fetal head, meconium-stained fluid and active uterine contractions.
**Day 0**	**Emergency Delivery and NICU Admission** Emergency cesarean section performed due to suspected chorioamnionitis, active labor and a large fetal tumor. Neonate born in critical condition (Apgar scores: 6 at 1 min, 6 at 3 min, 4 at 5 min, 4 at 10 min). Immediately intubated and admitted to the NICU. Examination confirmed a large sacrococcygeal mass.
**Day 1**	**Initial Diagnostic Workup and Imaging** Echocardiography: Identified Patent Foramen Ovale (PFO) and Patent Ductus Arteriosus (PDA)—physiological findings in the early postnatal period. Cranial Ultrasound: Revealed high-resistance Middle Cerebral Artery (MCA) flow. CT Abdomen/Pelvis: Revealed a voluminous, heterogeneous mixed solid-cystic sacrococcygeal tumor (120 × 85 × 120 mm) with neovascularization; liver metastases suspected.
**Days 1–3**	**Clinical Stabilization and Neonatal Care** Intensive medical stabilization was provided due to severe anemia, thrombocytopenia, and coagulopathy. Interventions: Administration of surfactant, broad-spectrum antibiotics, hemostatic management, and multiple blood product transfusions.
**Day 3**	**Surgical Resection and Reconstruction** Surgical Intervention: Complete en bloc resection of the 570 g sacrococcygeal tumor along with a coccygectomy. Successful anatomical reconstruction of the gluteal and anorectal regions.
**Day 6**	**Early Postoperative Imaging** Follow-up CT: Detected an enlarged left inguinal lymph node; no definitive distant metastases were visualized.
**Day 13**	**Postoperative Evaluation** Pelvic MRI: Demonstrated a small residual presacral cystic lesion, suspicious for minor residual tumor tissue. Wound Evaluation: Surgical incisions healed by primary intention without complications. Nutrition: Enteral feeding introduced and well-tolerated by the neonate.
**Day 34**	**Histopathology and Long-term Strategy** Final Pathology: Confirmed Grade 3 immature teratoma. Treatment Strategy: The oncology multidisciplinary team decided against chemotherapy due to a steadily falling Alpha-Fetoprotein (AFP) level and the absence of clear distant metastases. Protocol: Strict surveillance protocol initiated including serial imaging and tumor markers (AFP, β-hCG, LDH).
**Day 49**	**Extubation** Successfully extubated; infant breathing spontaneously with only passive oxygen support.
**Day 81**	**Hospital Discharge** Discharged home in good clinical condition at a weight of 2475 g. Discharge Plan: Scheduled for close, ongoing outpatient pediatric oncology follow-up.
**3 Months of Age**	**Infant Follow-Up and Surveillance Outcomes** Surveillance Imaging: Follow-up CT of the chest, abdomen, and pelvis confirmed no distant metastases. Laboratory Markers: Significant reduction in AFP levels from >121,000 ng/mL at birth down to 232 ng/mL. Functional Status: Infant is clinically well, showing appropriate growth, normal bowel function, and normal bladder function.

## Data Availability

The original contributions presented in this study are included in the article; further inquiries can be directed to the corresponding authors.
